# EST Analysis of *Ostreococcus lucimarinus,* the Most Compact Eukaryotic Genome, Shows an Excess of Introns in Highly Expressed Genes

**DOI:** 10.1371/journal.pone.0002171

**Published:** 2008-05-14

**Authors:** William Lanier, Ahmed Moustafa, Debashish Bhattacharya, Josep M. Comeron

**Affiliations:** 1 Interdisciplinary Program in Genetics, University of Iowa, Iowa, United States of America; 2 Department of Biological Sciences and Roy J. Carver Center for Comparative Genomics, University of Iowa, Iowa, United States of America; American Museum of Natural History, United States of America

## Abstract

**Background:**

The genome of the pico-eukaryotic (bacterial-sized) prasinophyte green alga *Ostreococcus lucimarinus* has one of the highest gene densities known in eukaryotes, yet it contains many introns. Phylogenetic studies suggest this unusually compact genome (13.2 Mb) is an evolutionarily derived state among prasinophytes. The presence of introns in the highly reduced *O. lucimarinus* genome appears to be in opposition to simple explanations of genome evolution based on unidirectional tendencies, either neutral or selective. Therefore, patterns of intron retention in this species can potentially provide insights into the forces governing intron evolution.

**Methodology/Principal Findings:**

Here we studied intron features and levels of expression in *O. lucimarinus* using expressed sequence tags (ESTs) to annotate the current genome assembly. ESTs were assembled into unigene clusters that were mapped back to the *O. lucimarinus* Build 2.0 assembly using BLAST and the level of gene expression was inferred from the number of ESTs in each cluster. We find a positive correlation between expression levels and both intron number (R = +0.0893, *p* = <0.0005) and intron density (number of introns/kb of CDS; R = +0.0753, *p* = <0.005).

**Conclusions/Significance:**

In a species with a genome that has been recently subjected to a great reduction of non-coding DNA, these results imply the existence of selective/functional roles for introns that are principally detectable in highly expressed genes. In these cases, introns are likely maintained by balancing the selective forces favoring their maintenance with other mutational and/or selective forces acting on genome size.

## Introduction

**Figure 1:**
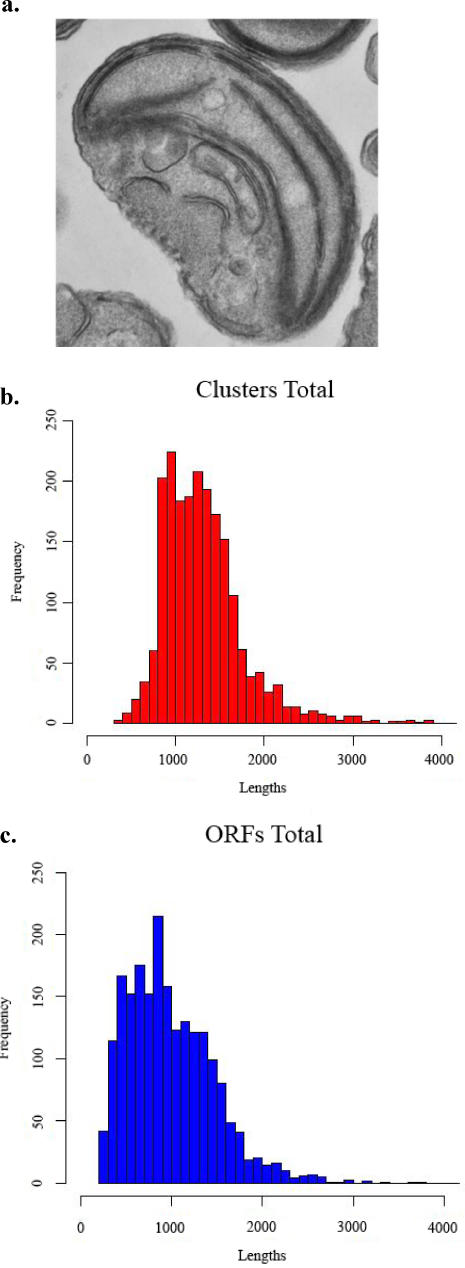
* Ostreococcus lucimarinus* and length distribution of clustered contigs and open reading frames. The prasinophyte green algal genus *Ostreococcus* is the smallest-known free-living eukaryotes, with an average size of 0.8 μm. (a) Image of *Ostreococcus* strain RCC 143 kindly provided by W. Eikrem and J. Throndsen (University of Oslo) (image also available in Wiki Commons). (b) and (c) Histograms showing the frequency and length distribution for clustered contigs (b) and longest open reading frames (c) for the *Ostreococcus lucimarinus* EST library.

The genus *Ostreococcus* encompasses a group of globally distributed photosynthetic, unicellular green algae in the anciently diverged class Prasinophyta [1]. These cells are the smallest known eukaryotes [2], with cell sizes smaller than 1 μm (Figure 1a). Among free-living taxa, *Ostreococcus* species also contain the most compact (i.e., gene-dense) genomes [3]. For example, in comparison to the compact genome of *Saccharomyces cerevisiae* (12.1 Mbp) that contains ∼6000 open reading frames, with one gene every 2.0 kb, *O. tauri* and *O. lucimarinus* have genomes sizes of 12.6 and 13.2 Mbp with a gene occurring every 1.6 and 1.7 kb, respectively [3]–[5]. This degree of genome miniaturization is unparalleled among free-living eukaryotes and has led to the contraction of intergenic regions to an average of less than 200 bp in *O. tauri*, which is the smallest among eukaryotes [6], [7]. Yet surprisingly, the dearth of noncoding DNA is not maintained across the *Ostreococcus* genomes because introns are present in an estimated 20–25% of transcripts, compared to less than 4% of intron-containing genes in the budding yeast [4], [5]. This algal genome structure is in sharp contrast to the general positive relationship between intron and intergenic size, and ultimately genome size, observed across a broad range of eukaryotes [7]. In fact, *Ostreococcus* species display the highest ratio of intragenic to intergenic noncoding DNA detected thus far.

This extreme level of genome compression has lead to both a downsizing of gene families and in some cases to the creation of gene fusion proteins [3], [4]. These trends are conserved across the *Ostreococcus* genomes which are characterized by a high degree of synteny for a core set of 18 chromosomes. Apart from this conservation, several chromosomes appear to have unique characteristics when compared both to other chromosomes and when compared across *Ostreococcus* spp. Chromosome 21 appears to be a unique feature of the *O. lucimarinus* genome resulting from a fusion of duplicated regions within Chromosomes 9 and 13 [4]. Chromosome 2, although displaying regions of synteny between species, has also been characterized as unique for its increased presence of non-coding DNA (intergenic and intronic) and transposable elements. Genes encoded on Chromosome 2 contain a higher proportion of shorter (40–65 bp) introns lacking canonical splice site and branch point sequences [3].

Despite these differences both within and between the *Ostreococcus* genomes, trends common to both suggest that similar mechanisms have led to the optimization of cell and genome size [4]. Thus the elevated presence of introns in these otherwise highly compact genomes, together with phylogenetic studies suggesting that this unusual genome structure is an evolutionarily derived state in prasinophytes [8], provides an unique opportunity to study evolutionary features associated with shifts in the amount of noncoding DNA and genome size. Here, we studied the possible role of natural selection acting on intronic DNA by investigating the influence of varying levels of expression on intron presence and length in *O. lucimarinus*. Our results reveal significant correlations between the levels of expression and the number of introns present in assembled EST clusters, strongly suggesting non-neutral processes. We present and discuss several possible selective mechanisms acting on intronic DNA focusing on explanations for intron retention in compact genomes.

## Methods

EST reads of *O. lucimarinus* that are available at the Joint Genome Institute (http://jgi.doe.gov/) were downloaded and assembled into unigene clusters using the TGI clustering package (http://www.tigr.org/software). Single contig clusters were mapped backed to the genome assembly of this species using blastn. Individual clusters which mapped to multiple loci and/or arose from cluster assemblies producing multiple contigs were omitted to reduce the mapping of clusters potentially containing EST reads of duplicate genes and closely related members of gene families.

Assembled clusters were verified as originating from a single locus by examining the clustered output for congruency within the directionality of forward and reverse EST reads. Clusters that contained forward or reverse reads in both the sense and antisense orientation of an assembled contig were examined to identify potential over-clustering within overlapping 3′ ends of transcripts. Over-clustering is not unexpected in *Ostreococcus* spp. due to the known presence of overlapping genes on opposite DNA strands in these species [4] . This procedure entailed identifying the presence of annotated protein models on both the sense and antisense orientations of the cluster. In cases where over-clustering was found, ESTs from overlapping loci were manually separated into sense and antisense bins, re-clustered independently, and tested for their ability to retrieve >90% of a single protein model prior to being designated as a unigene cluster. Individual over-clustered contigs that did not conform to these criteria were removed from the analysis. In addition, Chromosome 21 was omitted from the analysis because it may be a recent acquisition representing a chimera of Chromosomes 9 and 13 [4]. Finally, given the relatively high levels of expression and intron content of ribosomal proteins in eukaryotic genomes, the contribution of this class to overall trends in the data was assessed by querying the assembled clusters with all available nuclear encoded ribosomal models (n = 106) using blastx.

Tentative introns were identified as instances where complete and uninterrupted queries were not recovered with a single high-scoring pair (HSP). Intron presence was confirmed only in cases where non-overlapping HSPs, with an identity of 99% or greater, could be used to recover >97% of the query length. In cases where blastn was not capable of unambiguously aligning the terminal edges of exons, absolute intron size was calculated using the genomic distance between aligned neighboring HSPs. Adjustments to absolute intron size were performed by centering HSPs along their respective neighboring exons and stipulating the single inclusion of each query nucleotide in either one or the other HSP. Absolute intron size was therefore either decreased or increased in situations where terminal exon nucleotides were omitted or accounted for multiple times respectively in the local alignments. Statistical analyses of expression and intron composition were done using the JMP statistical software package (http://www.jmp.com/). Given the unique features of Chromosome 2 with respect to increased intron presence, we redid the analyses excluding this chromosome. Differences in the level of gene expression for Chromosome 2 were also compared against all other chromosomes to assess potential differences in gene expression and their influence on the correlations of expression and intron presence. Functional annotation of the 265 intron-containing clusters was performed using Blast2GO (www.blast2go.de).

## Results

To investigate the distribution of introns in *O. lucimarinus*, ESTs from this species were downloaded and assembled into unigene clusters (see Methods). Analysis of the 19,200 raw EST reads generated 2,050 clusters. The average cluster size was 7.57 ± 18.62 ESTs/cluster and there was an upper bound of several hundred reads and an under-representation of smaller transcripts (otherwise common to eukaryotic transcriptomes). These results are typical for size-selected and non-normalized cDNA libraries (Figures 1b, 1c). The BLAST results generated using nuclear encoded ribosomal proteins as the query returned 18.9% (20/107) of the working models within the clustered EST data (Table S1). As suggested by the distribution of the EST clusters in Figures 1b and 1c, the ribosomal models identified within the expression data were biased towards the identification of longer cDNAs with the shortest ribosomal protein models absent from the data set.

Investigation of potentially over-clustered ESTs due to overlapping transcriptional units on opposite strands that span small intergenic regions identified 8.9% (148/1647) of these cases (See Methods). Of these, only 26 clusters (15 are intronless and 11 contain introns) were found to contain either *Ostreococcus* models or data present in the NCBI non-redundant (nr) database in an orientation indicative of paired loci overlapping on opposite strands. Three of the intronless clusters were discarded from further analysis due to an extensive amount of overlap and the inability to properly bin the EST reads as belonging to one of the working models. Corrections of the starting clusters in this manner resulted in a final data set of 1,670 clusters for downstream analysis.

EST-cluster to genome alignments were used to map the 1,670 clusters to single loci at greater than 97% query coverage. The number of clusters mapped per chromosome was in most cases proportional to the number of annotated genes present in the individual chromosome scaffolds for *O. lucimarinus* Build 2.0 with an average of 22.4 ± 3.4% of annotated genes per chromosome within our mapped unigene clustered dataset (Table 1). Manual inspection of these clusters revealed 265 intron-containing clusters accounting for a total of 457 introns with an average size of 188 bp (Table 2). Forty-four of these clusters (16.7%) mapped to chromosome 2 and accounted for 181 (39.6%) of the introns (Table 1).

**Table 1:** Summary of genes predicted and EST-annotated (mapped) for O. lucimarinus.

Chr. #	Genes Predicted	Clusters mapped	Clusters with Introns	% Map/Predicted
1	684	146	19	21.4
2	489	136	46	27.8
3	589	149	19	25.2
4	524	117	14	22.3
5	492	115	17	23.4
6	454	83	12	18.3
7	463	111	10	24.0
8	426	91	15	21.4
9	414	85	13	20.5
10	358	73	17	20.4
11	328	68	10	20.7
12	325	68	10	20.9
13	298	76	7	25.5
14	373	82	14	21.9
15	267	46	7	17.2
16	262	52	11	19.9
17	209	50	10	23.9
18	79	26	1	32.9
19	92	20	4	21.7
20	330	76	9	23.0
Totals	7456	1670	265	22.4

Genes predicted for O. lucimarinus Build 2.0 in relation to the unigene clusters mapped, mapped clusters with introns, and the ratio of unigene clusters mapped per genes predicted in the currently genome assembly.

**Table 2:** Summary statistics of predicted introns in O. lucimarinus.

Average Intron Size, bp	188
Longest Intron, bp	1773
Shortest Intron, bp	26
Average Intron Number per transcript	1.74
Maximum Introns In Single transcript	13

Although our limited EST data address only ∼22% of the annotated genes (1,670/7,456 predicted genes), the coverage of these transcripts is largely unbiased in their chromosomal distribution. Furthermore, our analyses indicate a similar average intron size (188 bp) to that described for the complete genome assembly that utilized both experimental and bioinformatic methods to calculate this number (187 bp). This suggests the EST data utilized here do not deviate significantly from characteristics of the complete genome and therefore represent an accurate picture of the overall pattern of intron evolution in *O. lucimarinus*. Furthermore, analysis of expression patterns failed to find a difference in the levels of gene expression for clusters that mapped to intron-rich Chromosome 2 when compared to all other chromosomes (Mann-Whitney U-test; Z = 1.24, *p*<0.2157) suggesting that although different in intron composition, the levels of relative expression for the genes on Chromosome 2 are comparable to the remainder of the genome.

**Figure 2:**
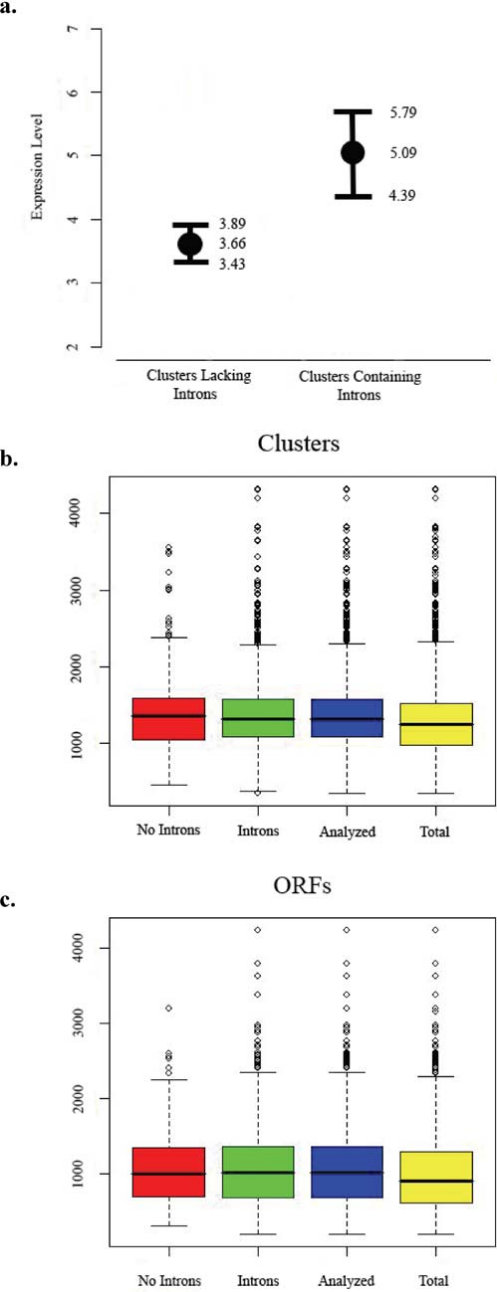
Levels of gene expression for intron containing and intronless genes in *Ostreococcus lucimarinus.* (a) Mean expression values and +/− standard errors for clusters lacking introns and all clusters containing introns. Expression values are in terms of the number of sequenced clones represented within a single unigene cluster. Boxplots showing the clustered contig lengths (b) and longest open reading frames (c) for contigs without introns (red, n = 265), contigs with introns (green, n = 1405), the combined data set of intron containing and intronless contigs (blue n = 1670), and for the complete EST library (yellow, n = 2050).

Nonparametric Spearman's rank correlation analysis using intron number in relation to the number of overlapping sequenced clones contained within unique gene clusters (used as a proxy for expression levels) returned a positive correlation between intron presence and increased expression (R = +0.0893, *p*<0.0005); an equivalent result is observed after excluding clusters mapped to Chromosome 2 (R = +0.0972, *p*<0.0005; see Table 3). Congruent with the Spearman's rank correlation, the comparison of intron-less and intron-containing genes showed significantly reduced expression for intron-less genes (Mann-Whitney U-test; Z = 3.95, *p*<0.0001; Figure 2a). Note that these results remain consistent if ribosomal proteins (n = 20) are excluded from the analysis, which results in a positive correlation between intron presence and expression levels (R = +0.0970, *p*<0.0005) and significantly reduced expression for intron-less genes when compared to intron-containing genes (Mann-Whitney U-test; Z = 4.18, *p*<0.0001). These latter results are not surprising given the limited contribution of ribosomal proteins to the EST library (Table S1; see Discussion).

**Table 3:** Spearman's rank correlation coefficient analysis of EST-annotated introns in O. lucimarinus.

	All chromosomes	Excluding Chr. 2	Chr.2 Only	Excluding ribosomal proteins
Sequenced Clones^1^ vs. Intron Number	0.0893 ***	0.0972 ***	0.0132 n.s.	0.0970 ***
Sequenced Clones vs. Intron Number/LORF^2^	0.0753 **	0.0839 **	−0.0300 n.s.	0.0833 **
Sequenced Clones vs. Mean Intron Length	0.1008 ***	0.0987 ***	0. 0459 n.s.	0.1019 ***
Sequenced Clones vs. Cluster Length	0. 4741 ***	0.4627 ***	0. 6049 ***	0.4764 ***
Sequenced Clones vs. LORF	0. 4771 ***	0.4687 ***	0. 5757 ***	0.4783 ***
Cluster Length vs. Intron Number	−0.0189 n.s.	−0.0130 n.s.	−0.0608 n.s.	−0.0103 n.s.

1 Number of sequenced clones within assembled unigene clusters used as a measure of gene expression.

2 Length of the open reading frame.

* , *p*<0.05;

** , *p*<0.005;

*** , *p*<0.0005;

n.s., *p*>0.05

Interestingly, gene expression was positively correlated with both assembled cluster length (R = 0.4741, *p<*0.0005) and corresponding longest open reading frame (R = 0.4771, *p<*0.0005) in all analyses. The tendency for EST libraries to be biased towards longer transcripts has been previously reported [9]. These studies suggest that the direct use of EST data might be problematic and that it should be normalized for length if transcript abundance is to be used as a measure of expression levels. If a standard normalization of expression is applied here (i.e., Normalized Expression = EST reads/Cluster Length [9]) the correlation between intron presence and increased expression is further strengthened (R = +0.1251, *p<*0.0001). In addition, no statistical differences were found between intron-containing or intron-less unigene clusters in terms of either the assembled cluster lengths or longest open reading frames (Figures 2b, 2c and Table 4). This distinction is important because it addresses the possibility of our results being generated from the bias of intron containing transcripts simply being longer than the intron-less transcripts.

**Table 4:** Summary statistics for cluster lengths and longest open reading frames (LORFs) for all unigene clusters.

	All Data	Intronless	Intronic	Intronic excluding Chr. 2
**Clusters**		n = 1405	n = 265	n = 219
Longest, bp	4309	4309	3502	3502
Shortest, bp	344	344	456	456
Mean, bp	1358	1361	1340	1351
Median, bp	1308	1304	1321	1335
σ^2^, bp	455	450	479	495
**LORFs**				
Longest, bp	4230	4230	3192	3192
Mean, bp	1059	1060	1052	1078
Median, bp	1007	1011	1005	1017
Shortest, bp	201	201	288	288
σ^2^, bp	492	497	463	465

On the other hand, previous studies conducted on the expression profiles of other highly compact eukaryotic genomes have noted that basic processes, such as transcription, are usually modified with a tendency for overlapping transcripts [10]. This issue was addressed in our study by examining the potential for over-clustered contigs using existing protein models and the directionality of clustered EST reads within individual contigs (See Methods). Summary statistics suggest that the effect of over-clustered loci residing within unique clusters is minimal (Table 4).

Lastly, the trend of intron presence and increased expression does not appear to be an artifact of longer transcripts simply containing more introns because a measure of intron density (number of introns/length ORF) is also positively associated with expression (R = +0.0753, *p<*0.005, R = +0.1196, *p<*0.0001 when corrected for length), whereas intron number and assembled cluster length was consistently found to be non-significant in all analyses. The removal of ribosomal proteins from the analysis depicts an equivalent trend, with intron density increasing with expression (R = +0.0833, *p<*0.005).

## Discussion

Attempts to model the evolution of genome size typically involve a tendency for the mutational process of small insertions and deletions (indels) to be biased towards deletions (deletion bias) that progressively eliminate nonessential portions of the genome. This observation offers a quandary in terms of intron retention in the reduced genomes of *Ostreococcus* [11]–[20]. Yet, unidirectional differences in deletion bias *alone* cannot account for the ratios of genome sizes and retention of non-coding sequence in all instances because these phenomena have been shown to not always correlate with observed patterns in genome size. This in turn suggests that genome sizes evolve out of a combination of mutational biases, neutral drift, and selection [21]–[23]. Hence recent models have focused on the balance between expansion and contraction of non-coding DNA due to both small and larger indels (i.e., repetitive elements) while including selective forces that might vary between non-coding sequences in an attempt to better characterize these effects in terms of the evolution of genome size [15], [21], [24].

Several biological arguments have previously been made to justify extreme directional shifts in genome size as a byproduct of selection working towards larger and smaller cell volumes. Examples of cell size and corresponding shifts in genome size have been proposed for gamete dispersal, duration of meiotic division, and responses to CO_2_ in plants [13]. These explanations however cannot explain differences between the preferential retention of particular non-coding sequences (i.e., intergenic vs. intronic) in cases where genome reduction has occurred as a result of either ‘random’ deletion biases or selective forces driving smaller cell volumes.

The analyses described here were aimed at investigating features of intron evolution and to provide a potential explanation for the disproportionate representation of introns in *O. lucimarinus* when compared to other non-coding regions of the genome. Interestingly, we find a positive correlation of intron presence and increased expression, which suggests a selective advantage (i.e., function) for introns in highly expressed genes that is not present (or present to a smaller degree) in genes with reduced expression. This finding is not unique to the miniaturized genome of *Ostreococcus* because it has previously been reported in yeast. In *S. cerevisiae*, a few intron-containing genes produce nearly a third of the total mRNA transcripts, with a large fraction of these intron-containing genes corresponding to ribosomal proteins [25]. Nevertheless, *Ostreococcus* represents a more extreme case in this respect because its genome is not only more gene dense but also clearly shows a much higher proportion of genes with introns. Furthermore, the reported relationships between expression and intron features are also observed after removing ribosomal proteins from the analyses in *Ostreococcus*. Indeed, several functional categories were represented by intron-containing genes with diverse roles in metabolism, responses to light, and chloroplast function (Table S2).

Potential explanations for intron presence (and retention) in highly expressed genes include their role in transcriptional elongation, nonsense-mediated mRNA decay (NMD), amelioration of linkage effects between neighboring sites under selection, and mRNA transport [26]–[31]. Although the relative contribution of these factors is unclear in this study, ultrastructural analysis of *Ostreococcus* has revealed the presence of nuclear pores in low number with an average pore density of 1–2 pores per nucleus [32]–[34]. This structural organization may represent a nuclear export bottleneck for *Ostreococcus*, thereby explaining stronger selection for intron maintenance in highly expressed genes as a result of the known increased export efficiency to the cytoplasm of spliced mRNAs [35].

Recent proteomic analyses of *Arabidopsis* nucleolus extracts have identified putative orthologs to exon-junction complex (EJC) proteins previously shown to increase the export of spliced mRNAs in animals [35], [36]. Using the tentative *Arabidopsis* EJC proteins identified in [36], we queried the existing *O. lucimarinus* protein models for the existence of these sequences in the current assembly (Table 5). Several of the *Arabidopsis* EJC protein families (AtALY, AtUAP56, AtDEK, and Atp15) converged on a single *Ostreococcus* model. Reciprocal BLAST analyses performed on these best candidate gene models using the nr protein data available at NCBI identified most gene models as their corresponding homologs through the conservation of protein domains. Of particular interest is the tentative identification of an ALY/REF homolog because this component has previously been characterized as an EJC interacting protein that is responsible for the enhanced export efficiency of spliced mRNAs [35]. The *Ostreococcus* nuclear pore complexes are never found to be facing the exterior membrane of the cell and are always directed towards the cell interior facing the chloroplast [34]. Even though the number of pores may be proportional to the reduced cell volume and small nucleus, this trait may impose a nuclear trafficking bottleneck for genes expressed at high levels. If correct, this cellular architecture and the potential existence of EJC proteins involved in the increased export of spliced mRNAs would help to explain the increased proportion of intron-annotated clusters involved in metabolism, responses to light, and chloroplast function (Table S2).

**Table 5:** Putative exon-junction associated proteins.

EJC Associated Proteins	*Arabidopsis* Locus	Model Protein ID	e-value
AtUAP56-1	At5g11220	43741	e-180
AtUAP56-2	At5g11170	43741	e-147
atALY-1	At5g59950	18962	5.00E-19
atALY-2	At5g02530	18962	1.00E-19
atALY-3	At5g66260	n/a	n/a
AtDEK-1	At3g48710	16371	3.00E-33
AtDEK-2	At5g63550	16371	2.00E-35
AtDEK-3	At4g26630	16371	6.00E-27
AtDEK-4	At5g55660	16371	1.00E-36
AtP15-1	At1g11570	13809	1.00E-15
AtP15-2	At1g27970	13809	6.00E-27
AtP15-3	At1g27310	13809	8.00E-26
AteIF-4AIII	At3g19760	39453	0
AtUPF2	At2g39260	37385	e-103
AtUPF3	At1g33980	16338	4.00E-06
AtMagoNashi	At1g02140	39734	3.00E-60
AtY14	At1g51510	32221	2.00E-32
AtRNPS1	At1g16610	n/a	n/a
AtSRm160	At2g29210	n/a	n/a

Putative exon-junction associated proteins in *Arabidopsis*, their corresponding locus, best hit to protein model in the *Ostreococcus lucimarinus* Build 2.0, and respective e-value. The proposed *Arabidopsis* EJC protein families have been grouped together.

Although it is to be expected that not all introns currently existing in the genome are of functional significance, the correlations described here are consistent with other findings that correlate intron features and increased levels of gene expression in plants. Analyses of rice and *Arabidopsis* genomes have revealed that highly expressed genes not only contain more introns but the average intron length and primary transcript length also increase with increasing expression [37]. The results reported here for an anciently diverged (albeit derived) green alga lead us to suggest that similar trends may occur in other plant and algal genomes.

Analyses done on animal models however have reported a more diverse pattern when investigating relationships between expression levels and intron size and density. Studies in *Drosophila melanogaster, Caenorhabditis elegans*, and humans suggest that intron size decreases with expression, opposite to the trend observed in plants [28], [38]–[41]. Results regarding intron density vary among studies likely reflecting differences in the methods used to infer levels of expression; i.e., cDNA microarray data, EST counts (uncorrected or normalized) or indices of synonymous codon bias used as an indirect approximation of expression levels. Expression studies done using uncorrected data from EST-libraries or indexes of codon bias [38], [39] are highly problematic and most likely biased when investigating intron density because both measures are strongly influenced by the length of the coding sequence [9], [28], [42]. More recent analyses using estimates of expression based on the more accurate normalized EST or cDNA microarray data suggest increased intron density with expression in both humans and *Drosophila* [28], [39] whereas *C. elegans* exhibits the opposite trend [39]. Thus, current data are consistent with intron density being positively correlated with gene expression in all lineages studied. In contrast, there are conflicting trends with regard to intron length and gene expression in plants and animals. This latter issue merits additional investigation.

Finally, it should be also noted that differences exist between the absolute numbers of intron containing transcripts reported here and those previously reported for the *O. lucimarinus* genome (16% vs. 20%) [3], [4]. This discrepancy likely arises from differences residing within the automated pipeline used for the annotation of introns within genes in the absence of EST support and/or as a result of a very limited or biased (e.g., size selection) sample of ESTs. This problem may be exacerbated by poorly conserved splice-sites and the absence of clear branch-point motifs [4]. We believe that the use of loci for which experimental data exist is particularly important when analyzing genomes with a highly derived and complex pattern of noncoding DNA, as is *Ostreococcus* and other pico-eukaryotes.

Although our study is consistent with previously documented intron composition trends seen in other plant genomes, it clearly underscores the existence of selective (and likely functional) roles of introns in genes that are highly expressed in *O. lucimarinus*. A note of caution is that current EST data for this species are rather limited and future experiments are needed to fully corroborate our results, using more complete expression data from *Ostreococcus* spp. This work would allow us to further explore the potential contribution that introns make in reduced genomes of free-living picoeukaryotes.

## Supporting Information

Table S1 BLAST results generated using nuclear encoded ribosomal proteins within the clustered EST data
(0.05 MB DOC)

Table S2 BLAST2GO annotated intron clusters
(0.14 MB DOC)
