# Supplementary material for: EST Analysis of Ostreococcus lucimarinus, the Most Compact Eukaryotic Genome, Shows an Excess of Introns in Highly Expressed Genes
Source: PLoS One. 2008 May 14;3(5):e2171. doi: 10.1371/journal.pone.0002171 (PMC2367439; doi:10.1371/journal.pone.0002171)
Supplement: Table S1 — BLAST results generated using nuclear encoded ribosomal proteins within the clustered EST data (0.05 MB DOC) [file pone.0002171.s001.doc]

**Table S1.** BLAST results generated using nuclear encoded ribosomal proteins within the clustered EST data.

| **Annotation** | **Ribosomal Protein Model (Query)** | **Unigene Cluster (Database)** | **% identity** | **% coverage** | **Sequenced Clones** | **e-values** |
| --- | --- | --- | --- | --- | --- | --- |
| 60S L22 | estExt_fgenesh1_pg.C_Chr_140175|Ost9901_3 | CL83Contig1 | 100 | 62.8 | 15 | 0 |
| RPL4/L1e | estExt_Genewise_ext.C_Chr_150094|Ost9901_3 | CL1651Contig1 | 99.7 | 86.5 | 1 | 4E-162 |
| L3 | estExt_fgenesh1_pg.C_Chr_10038|Ost9901_3 | CL292Contig1 | 100 | 99.7 | 5 | 0 |
| RPPO | eugene.2000010013|Ost9901_3 | CL943Contig1 | 100 | 99.7 | 2 | 7E-154 |
| RPL5A | estExt_Genewise_ext.C_Chr_140161|Ost9901_3 | CL362Contig1 | 100 | 93.7 | removed | 3E-143 |
| RPSa | fgenesh1_pm.C_Chr_3000075|Ost9901_3 | CL1297Contig1 | 100 | 99.7 | 1 | 1E-143 |
| RPS3a | eugene.1900010014|Ost9901_3 | CL774Contig1 | 100 | 87.7 | 2 | 4E-129 |
| RPL8 | eugene.0400010192|Ost9901_3 | CL1354Contig1 | 100 | 98.4 | 1 | 7E-132 |
| RPL7a | e_gwEuk.6.445.1|Ost9901_3 | CL946Contig1 | 100 | 98.0 | 2 | 1E-102 |
| RPS6 | gwEuk.3.536.1|Ost9901_3 | CL1848Contig1 | 99.6 | 100.0 | 1 | 1E-90 |
| RPL10a | estExt_GenewiseEukaryote.C_Chr_40517|Ost9901_3 | CL1909Contig1 | 100 | 99.5 | 1 | 2E-106 |
| RPL19 | estExt_fgenesh1_pg.C_Chr_20155|Ost9901_3 | CL976Contig1 | 100 | 70.5 | 2 | 2E-57 |
| RPL9 | estExt_GenewiseEukaryote.C_Chr_40538|Ost9901_3 | CL1244Contig1 | 99.5 | 99.5 | 1 | 7E-106 |
| RPL6 | eugene.0900010363|Ost9901_3 | CL572Contig1 | 99.5 | 98.9 | 3 | 1E-90 |
| RPL23 | eugene.0400010167|Ost9901_3 | CL352Contig1 | 100 | 99.5 | 4 | 8E-23 |
| RPL17 | eugene.0600010293|Ost9901_3 | CL1012Contig1 | 100 | 99.5 | 2 | 2E-89 |
| RPL27aB | eugene.1400010117|Ost9901_3 | CL1925Contig1 | 100 | 99.3 | 1 | 9E-76 |
| RPS18 | eugene.2000010297|Ost9901_3 | CL954Contig1 | 100 | 96.6 | 2 | 2E-72 |
| RPS16 | eugene.0500010393|Ost9901_3 | CL1311Contig1 | 100 | 99.3 | 1 | 7E-66 |
| RPS26 | fgenesh1_pg.C_Chr_2000307|Ost9901_3 | CL1475Contig1 | 100 | 99.1 | 1 | 1E-39 |
