# Supplementary material for: EST Analysis of Ostreococcus lucimarinus, the Most Compact Eukaryotic Genome, Shows an Excess of Introns in Highly Expressed Genes
Source: PLoS One. 2008 May 14;3(5):e2171. doi: 10.1371/journal.pone.0002171 (PMC2367439; doi:10.1371/journal.pone.0002171)
Supplement: Table S2 — BLAST2GO annotated intron clusters (0.14 MB DOC) [file pone.0002171.s002.doc]

**Table S2.** BLAST2GO annotated intron clusters.

| TGI Assembled Intron Containing Unigene Cluster | BLAST2GO Annotation |
| --- | --- |
| CL1022Contig1 | Ostreococcus lucimarinus predicted protein |
| CL1132Contig1 | Ostreococcus lucimarinus predicted protein |
| CL1153Contig1 | Ostreococcus lucimarinus predicted protein |
| CL1188Contig1 | Ostreococcus Chr2 scafold |
| CL1233Contig1 | Ostreococcus lucimarinus predicted protein |
| CL1237Contig1 | Ostreococcus lucimarinus predicted protein |
| CL1267Contig1 | Ostreococcus lucimarinus predicted protein |
| CL127Contig1 | Ostreococcus lucimarinus predicted protein |
| CL1280Contig1 | Ostreococcus lucimarinus CCE9901 Nucleic acid binding GTPase, translation factor, putative YchF |
| CL1293Contig1 | Ostreococcus lucimarinus predicted protein |
| CL1332Contig1 | Ostreococcus lucimarinus predicted protein |
| CL1340Contig1 | Ostreococcus lucimarinus predicted protein |
| CL135Contig1 | Ostreococcus lucimarinus predicted protein |
| CL1481Contig1 | Ostreococcus lucimarinus predicted protein |
| CL162Contig1 | Ostreococcus lucimarinus predicted protein |
| CL1634Contig1 | Ostreococcus lucimarinus predicted protein |
| CL1659Contig1 | Ostreococcus lucimarinus predicted protein |
| CL1688Contig1 | Ostreococcus lucimarinus predicted protein |
| CL1689Contig1 | Ostreococcus lucimarinus predicted protein |
| CL168Contig1 | Ostreococcus lucimarinus predicted protein |
| CL1758Contig1 | psbX, PSII-X, photosystem II |
| CL1779Contig1 | Ostreococcus lucimarinus predicted protein |
| CL1789Contig1 | Ostreococcus lucimarinus predicted protein |
| CL1859Contig1 | Ostreococcus lucimarinus predicted protein |
| CL1957Contig1 | Ostreococcus lucimarinus predicted protein |
| CL196Contig1 | Ostreococcus lucimarinus predicted protein |
| CL307Contig1 | Ostreococcus lucimarinus predicted protein |
| CL319Contig1 | Ostreococcus lucimarinus predicted protein |
| CL352Contig1 | Ribosomal protein l23 |
| CL356Contig1 | Ostreococcus lucimarinus predicted protein |
| CL539Contig1 | Annexin family transporter: Annexin, A13 (OSTLU_6061) |
| CL543Contig1 | MC family transporter: phosphate (OSTLU_25740) |
| CL589Contig1 | Ostreococcus lucimarinus predicted protein |
| CL659Contig1 | Ostreococcus lucimarinus predicted protein |
| CL748Contig1 | Ostreococcus lucimarinus predicted protein |
| CL816Contig1 | Ostreococcus lucimarinus predicted protein |
| CL833Contig1 | Ostreococcus lucimarinus predicted protein |
| CL850Contig1 | Ostreococcus lucimarinus predicted protein |
| CL87Contig1 | geranylgeranyl reductase (Ggh) |
| CL883Contig1 | photosystem I light harvesting complex, chlorophyll a/b binding (lhca3) |
| CL8Contig1 | Ostreococcus lucimarinus predicted protein |
| CL905Contig1 | Ostreococcus lucimarinus predicted protein |
| CL917Contig1 | Ostreococcus lucimarinus predicted protein |
| CL918Contig1 | Ostreococcus lucimarinus predicted protein |
| CL92Contig1 | Ostreococcus lucimarinus predicted protein |
| CL932Contig1 | ZIP family transporter: zinc ion (OSTLU_29694) |
| CL934Contig1 | psaD, PSI-D, subunit II, photosystem I protein (psaD) |
| CL957Contig1 | homogentisate phytylprenyltransferase/homogentisic acid geranylgeranyl transferase (Hpt1) |
| CL964Contig1 | Ostreococcus lucimarinus predicted protein |
| CL975Contig1 | Ostreococcus lucimarinus predicted protein |
| CL981Contig1 | Ostreococcus lucimarinus predicted protein |
| CL98Contig1 | Ostreococcus lucimarinus predicted protein |
| CL9Contig1 | Ostreococcus lucimarinus predicted protein |
| CL1010Contig1 | adp-glucose pyrophosphorylase large subunit |
| CL1017Contig1 | myb family transcription factor |
| CL1063Contig1 | transcription regulator |
| CL107Contig1 | glutaminecatalytic region |
| CL1114Contig1 | heme activated protein |
| CL1115Contig1 | dna mismatch repair protein |
| CL1131Contig1 | related cyclin family protein |
| CL1143Contig1 | ubiquinol--cytochrome-c reductase-like protein |
| CL114Contig1 | nitrate reductase |
| CL1151Contig1 | photosystem i subunit iii |
| CL115Contig1 | eukaryotic initiation factor 4a |
| CL116Contig1 | heat shock protein 90 |
| CL1171Contig1 | glutamine amidotransferase |
| CL118Contig1 | alcohol dehydrogenase class iii |
| CL1201Contig1 | 40s ribosomal protein s30 |
| CL1212Contig1 | dsba-like thioredoxin family protein |
| CL1219Contig1 | sjchgc05795 protein |
| CL1221Contig1 | acetylglutamate kinase |
| CL1250Contig1 | deoxyribonucleoside kinase |
| CL1256Contig1 | thylakoid lumenal 20 kda |
| CL1262Contig1 | heat shock protein hsp20 |
| CL1287Contig1 | glucokinase |
| CL1291Contig1 | clathrin assembly protein ap19 homolog |
| CL1297Contig1 | 40s ribosomal protein sa (laminin receptor-like protein) |
| CL1316Contig1 | protein phosphatasecatalyticbeta isoform |
| CL1354Contig1 | 60s ribosomal protein l8 |
| CL1366Contig1 | shoot1 protein |
| CL1383Contig1 | serine carboxypeptidase iii precursor |
| CL138Contig1 | chlorophyll a b-binding |
| CL1396Contig1 | phd finger |
| CL1452Contig1 | 50s ribosomal protein l15 |
| CL1459Contig1 | chromosome segregation protein smc |
| CL145Contig1 | phenylalanine-trna synthetase |
| CL1461Contig1 | nadh dehydrogenase |
| CL1469Contig1 | keratin filamentisoform cra_a |
| CL146Contig1 | protein phosphatase type-2c |
| CL1472Contig1 | integral membrane yip1 family protein |
| CL1495Contig1 | transcription factor e2f |
| CL149Contig1 | ferredoxin-nadp+ reductase |
| CL14Contig1 | elongation factor 3 |
| CL1505Contig1 | replication factor c (activator 1) 5 |
| CL1508Contig1 | presenilin family protein |
| CL1509Contig1 | 50s ribosomal protein l1 |
| CL1523Contig1 | 26s proteasome regulatory particle triple-a atpase subunit4 |
| CL1533Contig1 | dek oncogene (dna binding) |
| CL1545Contig1 | wd repeat domain 12 |
| CL1574Contig1 | inositol phosphatase-like protein |
| CL1579Contig1 | nuclear antigen 21d7 |
| CL1581Contig1 | histone deacetylase superfamily |
| CL1590Contig1 | nucleolar protein expressed |
| CL1592Contig1 | er-golgi snare complex subunit |
| CL160Contig1 | indole-3-glycerol phosphate synthase |
| CL1618Contig1 | peptidyl-prolyl cis-trans isomerase |
| CL1639Contig1 | single-stranded nucleic acid binding r3h |
| CL163Contig1 | nitrate transporter |
| CL1651Contig1 | 60s ribosomal |
| CL1655Contig1 | octicosapeptide phox bem1pdomain-containing protein tetratricopeptide repeat-containing protein |
| CL1687Contig1 | cop9 constitutive photomorphogenic homolog subunit 2 |
| CL1690Contig1 | transcription elongation factor spt4 |
| CL1692Contig1 | 3-phosphoinositide-dependent protein kinase-1 |
| CL1696Contig1 | sarcosine-dimethylglycine methyltransferase |
| CL1723Contig1 | fibrillarin 2 |
| CL1728Contig1 | molecular chaperone |
| CL1739Contig1 | ump synthase |
| CL173Contig1 | mg-protoporyphyrin ix chelatase |
| CL1754Contig1 | 50s ribosomal protein l19 |
| CL1769Contig1 | map2b (methionine aminopeptidase 2b) methionyl aminopeptidase |
| CL176Contig1 | octicosapeptide phox bem1pdomain-containing protein tetratricopeptide repeat-containing protein |
| CL1783Contig1 | oxygen evolving enhancer 2 of photosystem ii |
| CL1786Contig1 | dutp pyrophosphatase |
| CL1793Contig1 | protein phosphataseregulatory subunit b (pr 53) |
| CL1812Contig1 | xnop56 protein |
| CL1822Contig1 | wd40 repeat protein |
| CL1825Contig1 | fe-s metabolism associated |
| CL183Contig1 | fructose-bisphosphateclass ii |
| CL1848Contig1 | ribosomal protein s6 |
| CL184Contig1 | 3-keto-acyl-thiolase 2 |
| CL1855Contig1 | dicarboxylate tricarboxylate carrier |
| CL1877Contig1 | dna gyrase subunit a |
| CL1882Contig1 | 50s ribosomal protein l13 |
| CL188Contig1 | x-prolyl aminopeptidase (aminopeptidase p)soluble |
| CL1893Contig1 | tpa_inf: chloroplast light-harvesting complex i protein precursor lhca9 |
| CL1903Contig1 | protein kinase |
| CL1916Contig1 | riboflavin biosynthesis |
| CL1925Contig1 | ribosomal protein l27a |
| CL1926Contig1 | dead box atp-dependent rna helicase |
| CL192Contig1 | rna helicase |
| CL194Contig1 | beta-ketoacyl-acp synthase i |
| CL19Contig1 | fructose bisphosphate aldolase |
| CL2020Contig1 | ubiquitin-activating enzyme e1c |
| CL203Contig1 | dna polymerase lambda |
| CL232Contig1 | replication protein a 70kda |
| CL238Contig1 | enolase |
| CL247Contig1 | chaperonin 60 |
| CL255Contig1 | histidinol-phosphate aminotransferase |
| CL260Contig1 | at3g46740 t6h20_230 |
| CL269Contig1 | triosephosphate isomerase |
| CL27Contig1 | solute carrier familymember 5 |
| CL280Contig1 | myb family transcription factor |
| CL281Contig1 | calcium-dependent protein kinase |
| CL282Contig1 | chloroplast rna binding protein precursor |
| CL283Contig1 | constans-like b-box zinc finger protein |
| CL284Contig1 | 1-deoxy-d-xylulose 5-phosphate synthase |
| CL295Contig1 | the zys1a and b genes are linked in inverted orientation within a 15 kb genomicp213-~zys1a genomic sequence in ddbj accession number ab001485 |
| CL298Contig1 | zgc:152746 protein |
| CL308Contig1 | protein kinase |
| CL315Contig1 | swi snfmatrixactin dependent regulator ofsubfamilymember 5 |
| CL324Contig1 | muscleblind-like 3 |
| CL330Contig1 | possible fe2-oxoglutarate-dependent dioxygenase |
| CL334Contig1 | gamma tubulin |
| CL346Contig1 | plastid division protein |
| CL350Contig1 | protein kinase |
| CL354Contig1 | mgc53673 protein |
| CL355Contig1 | transcription initiation factorsubunit bdf1 and related bromodomain proteins |
| CL371Contig1 | 26s proteasome regulatory particle triple-a atpase subunit6 |
| CL38Contig1 | hb-1 (homeobox-1) transcription factor |
| CL390Contig1 | suppressor of variegation 3-9 homolog 2 |
| CL391Contig1 | gdp-mannose-dehydratase |
| CL395Contig1 | acetolactate synthase small |
| CL402Contig1 | possible conserved eukaryotic alpha beta hydrolase |
| CL403Contig1 | delta-aminolevulinic acid dehydratase |
| CL405Contig1 | solute carrier familymember b3 |
| CL406Contig1 | 3-methyl-2-oxobutanoate hydroxymethyltransferase |
| CL410Contig1 | cog0484:-class molecular chaperone with c-terminal zn finger domain |
| CL415Contig1 | chlorophyll a b-binding protein cp29 |
| CL428Contig1 | rna recognition motif-containing protein |
| CL42Contig1 | chaperonin 60 betachloroplast |
| CL431Contig1 | dna binding transcription factor |
| CL454Contig1 | heat shock protein |
| CL467Contig1 | holocarboxylase synthetase 2 |
| CL471Contig1 | chloroplast precursor |
| CL479Contig1 | tetrahydrofolate dehydrogenase |
| CL481Contig1 | vesicle-associated membranesynaptobrevin 7b |
| CL482Contig1 | mitochondrial f1-gamma subunit |
| CL487Contig1 | bromodomain transcription |
| CL492Contig1 | histone h3 |
| CL502Contig1 | protein kinase |
| CL50Contig1 | heat shock protein |
| CL510Contig1 | shaggy-related protein kinase kappa ask-kappa |
| CL517Contig1 | syntaxin 71-like protein |
| CL519Contig1 | atp-dependent clpproteolytic subunit |
| CL526Contig1 | chlorophyll a b-binding protein |
| CL533Contig1 | cyclin d1 |
| CL546Contig1 | ran binding protein |
| CL548Contig1 | histone h2a |
| CL551Contig1 | tata-box binding protein |
| CL553Contig1 | kda class i heat shock protein |
| CL554Contig1 | thioredoxin family protein |
| CL557Contig1 | porin-like protein |
| CL559Contig1 | quinone oxidoreductase |
| CL566Contig1 | developmentally regulated gtp binding protein 1 |
| CL569Contig1 | response regulator receiver domain protein |
| CL571Contig1 | possiblepsii-photosystem ii polypeptide |
| CL573Contig1 | cdc2-like protein kinase |
| CL576Contig1 | centromere microtubule binding protein cbf5 |
| CL59Contig1 | adenine nucleotide translocase |
| CL603Contig1 | histone acetyltransferase |
| CL604Contig1 | viral a-type inclusion |
| CL614Contig1 | aspartate aminotransferase |
| CL620Contig1 | chaperonin containingsubunit 6a (zeta 1) |
| CL625Contig1 | casein kinase ii beta chain ckb2 |
| CL626Contig1 | gtp-binding protein |
| CL631Contig1 | 30s ribosomal protein s1 |
| CL642Contig1 | glutamyl-trna reductase |
| CL660Contig1 | dihydrolipoamide dehydrogenase |
| CL66Contig1 | mitochondrial carrier protein |
| CL67Contig1 | glutathione reductase |
| CL682Contig1 | coatomer delta subunit |
| CL686Contig1 | phytanoyl-dioxygenasefamily protein |
| CL68Contig1 | telomere binding protein |
| CL705Contig1 | rnase p subunit p30 familyexpressed |
| CL710Contig1 | possible early light inducible protein |
| CL714Contig1 | phosphoribosyl pyrophosphate synthetase |
| CL719Contig1 | photosystem i subunit xi |
| CL732Contig1 | thylakoid lumenal 30 kdaprobable |
| CL736Contig1 | u4 u6-associated rna splicing factor |
| CL743Contig1 | angio-migratory cell protein |
| CL747Contig1 | snf7-like protein |
| CL749Contig1 | y4115_arath uncharacterized proteinchloroplast precursor |
| CL756Contig1 | adp-glucose pyrophosphorylase small subunit |
| CL763Contig1 | eukaryotic translation initiation factor 3 subunit 6 interacting protein |
| CL765Contig1 | unc-45 homolog a |
| CL767Contig1 | h+-transporting atp synthase-like protein |
| CL770Contig1 | asparagine synthetase b |
| CL774Contig1 | s-phase-specific ribosomal protein |
| CL790Contig1 | heat shock protein hsp20 |
| CL79Contig1 | glycine hydroxymethyltransferase |
| CL802Contig1 | ankyrin unc44 |
| CL806Contig1 | beta adaptin-like protein |
| CL80Contig1 | atp synthase gamma-subunit |
| CL817Contig1 | delta 9 desaturase |
| CL820Contig1 | glucose-6-phosphate phosphate translocator |
| CL821Contig1 | ribonucleotide reductase small subunit |
| CL822Contig1 | ribosomal protein l6 |
| CL824Contig1 | atp-dependent clp protease proteolytic subunit |
| CL828Contig1 | ribosomal protein l21 |
| CL845Contig1 | zinc finger |
| CL858Contig1 | ubiquitin conjugating enzyme |
| CL859Contig1 | protein phosphatase 2c |
| CL861Contig1 | rna-metabolising metallo-beta-lactamase |
| CL86Contig1 | abcfamily transporter: whiteprotein |
| CL875Contig1 | s-adenosyl-methionine-sterol-c- methyltransferase |
| CL895Contig1 | phosphatidylinositol phophatidylcholine transfer protein |
| CL919Contig1 | armadillo repeat containing 6 |
| CL929Contig1 | cyclin a |
| CL943Contig1 | 60s acidic ribosomal protein p0 |
| CL944Contig1 | domaink-homology type rna binding proteins |
| CL950Contig1 | at5g64760 mvp7_9 |
| CL967Contig1 | abc transporter protein |
